# The Role of Artificial Intelligence in Medication Management for Older Adults: A Systematic Review

**DOI:** 10.1002/agm2.70074

**Published:** 2026-04-26

**Authors:** Dipak Chandra Das, Moustaq Karim Khan Rony, Shovit Dutta, MD. Sami Al Zubair Zujbe, Tapan Bhattacharjee, Niloy Debnath, Shabbir Abdullah Maruf, Chowdhury Galib Mortuza, Farhana Rahman, Akash Das

**Affiliations:** ^1^ Public Health Foundation Bangladesh Dhaka Bangladesh; ^2^ Miyan Research Institute International University of Business Agriculture and Technology Dhaka Bangladesh; ^3^ Chattogram City Corporation Memon Maternity Hospital Chittagong Bangladesh; ^4^ Jamalpur Medical College Jamalpur Bangladesh; ^5^ Shahjalal University of Science and Technology Sylhet Bangladesh; ^6^ Chittagong Medical College Chittagong Bangladesh; ^7^ Bangladesh University of Professionals Dhaka Bangladesh; ^8^ Bangladesh University of Health Sciences Dhaka Bangladesh; ^9^ Shanto‐Mariam University of Creative Technology Dhaka Bangladesh; ^10^ Gonoshasthaya Samaj Vittik Medical College Dhaka Bangladesh

**Keywords:** artificial intelligence, digital health technology, geriatric care, medication management, older adults

## Abstract

Older adults face increased risks of medication non‐adherence, adverse drug events, and polypharmacy due to chronic health conditions and complex drug regimens. Traditional medication management approaches often fall short in addressing these challenges. Artificial intelligence (AI) has emerged as a promising tool for enhancing medication safety and personalization in geriatric care. This systematic review aimed to explore the role of AI in medication management for older adults, highlighting its effectiveness, usability, ethical implications, and integration within healthcare systems. Following PRISMA guidelines, a comprehensive search was conducted across six databases (PubMed, Scopus, Web of Science, CINAHL, IEEE Xplore, and Cochrane Library) for studies published between January 2015 and March 2025. Eligible studies included qualitative, quantitative, and mixed‐methods research on AI‐based medication interventions for individuals aged 60 and above. Data were synthesized thematically. Twenty‐nine studies were included. Five major themes emerged: (1) AI's ability to enhance adherence through smart reminders and automation; (2) personalized and predictive capabilities in managing complex regimens; (3) design and usability challenges among older adults; (4) ethical concerns related to trust, privacy, and autonomy; and (5) the importance of seamless integration within clinical workflows. Cross‐cutting observations emphasized the need for hybrid care models, inclusive design, and digital literacy training. AI has the potential to transform geriatric medication management. However, its success depends on ethical implementation, user‐centered design, healthcare integration, and attention to equity. Long‐term evaluations are essential to ensure sustainable and inclusive outcomes.

## Graphical abstract

Artificial intelligence enhances medication management for older adults by improving adherence, personalizing treatment, and predicting risks. Despite benefits, challenges remain in usability, trust, ethics, and system integration. Successful adoption requires user‐centered design, ethical safeguards, and seamless healthcare integration to ensure safe, equitable, and sustainable outcomes in geriatric care.

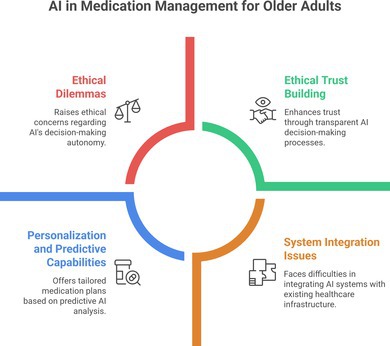

## 1 Introduction

The steady rise in the global population of older adults has become a defining demographic trend of the 21st century, with profound implications for healthcare delivery systems [1]. Individuals aged 60 years and above are living longer but often contend with multiple chronic illnesses, leading to complex medication regimens and increased vulnerability to adverse drug events [2]. Medication non‐adherence, dosage errors, and inappropriate polypharmacy are particularly prevalent among older adults, posing significant risks to their safety and well‐being [3]. Older adults are particularly vulnerable to medication‐related harm due to polypharmacy and age‐related physiological changes, increasing the likelihood of exposure to potentially inappropriate medications (PIMs) [4]. PIMs refer to drugs for which the risks outweigh the clinical benefits in older populations, particularly when safer alternatives exist or when medications exacerbate comorbid conditions or functional decline [5]. Traditional approaches to medication management have proven insufficient in mitigating these challenges, highlighting the urgent need for innovative, scalable solutions [6]. Within this landscape, artificial intelligence (AI) has emerged as a transformative force, offering tools and systems capable of enhancing medication safety, personalizing pharmacological care, and streamlining the work of healthcare providers [7].

Artificial intelligence, as applied to healthcare, encompasses a broad range of technologies including machine learning, natural language processing, and neural networks [8]. These tools have shown promise in detecting drug interactions, predicting adverse drug events, and supporting clinical decision‐making by processing vast volumes of patient data with speed and precision [9]. In the context of medication management for older adults, AI‐enabled tools such as smart pill dispensers, conversational agents, and predictive analytics platforms can help address adherence issues, optimize dosing, and reduce preventable medication‐related complications [10]. Importantly, these tools do not operate in isolation; rather, their efficacy often depends on thoughtful integration into existing healthcare workflows, user‐centered design, and alignment with the social and cognitive needs of older patients [11]. The growing body of research exploring these applications provides valuable insights into both the potential and the pitfalls of AI implementation in geriatric care [12].

However, the promise of AI in medication management should be weighed against a spectrum of practical and ethical concerns [13]. While AI can enhance precision and efficiency, older adults often face barriers to engaging with technology, ranging from low digital literacy to physical or cognitive limitations that impair usability [14]. Additionally, trust remains a critical issue: older individuals and their caregivers may be skeptical about allowing AI to influence or automate key health decisions, particularly in the absence of human oversight [15]. Concerning data privacy, transparency of algorithms, and the depersonalization of care further complicate the adoption landscape [16]. For healthcare professionals, the integration of AI can disrupt established workflows and require new forms of training and support [17]. These challenges underscore the need to view AI not merely as a technical solution, but as a socio‐technical innovation that should be carefully adapted to the realities of aging populations and healthcare systems [18].

Unlike prior reviews that often limit their scope to studies published before 2023 [1, 10], this systematic review incorporates research up to March 2025. This extended coverage enables the inclusion of recent innovations such as large language model (LLM)‐based prescription recognition and AI‐driven polypharmacy risk detection tools, providing a more up‐to‐date synthesis of evidence. Furthermore, this study does not narrowly focus on technical efficacy but holistically integrates ethical trust, socio‐cultural barriers, and hybrid care models as thematic pillars. This broader lens offers a more comprehensive understanding of AI's real‐world applicability in geriatric medication management, linking technological capability with social determinants of adoption. Therefore, this systematic review aims to critically examine the role of artificial intelligence in medication management for older adults.

## 2 Methodology

### 2.1 Review Design

This systematic review was conducted to explore and synthesize the existing body of qualitative literature on how artificial intelligence (AI) contributes to medication management among older adults. The review was guided by a structured and transparent process to ensure comprehensiveness and replicability, following the Preferred Reporting Items for Systematic Reviews and Meta‐Analyses (PRISMA) guidelines [19]. The protocol for this review was registered with the International Prospective Register of Systematic Reviews (PROSPERO) under the registration number CRD420251001155. A qualitative evidence synthesis approach was adopted to accommodate the exploratory and contextual nature of the research focus, allowing for a nuanced understanding of stakeholder perspectives, system usability, and implementation dynamics in real‐world healthcare settings. The primary research question guiding this review was: “How is artificial intelligence being utilized to support and improve medication management among older adults?”

### 2.2 Eligibility Criteria

Studies were selected based on their relevance to the review topic and their potential contribution to a thematic synthesis of findings. Eligible studies focused on individuals aged 60 years and older or on healthcare professionals' experiences in supporting medication management for this population using AI‐based tools. Peer‐reviewed original research articles employing qualitative, quantitative, or mixed‐methods designs were included, provided they contributed rich, descriptive data relevant to thematic analysis. Included studies examined AI‐driven interventions or technologies aimed at enhancing, supporting, or optimizing medication management, such as improving adherence, enabling personalized dosing, ensuring prescription accuracy, or monitoring adverse drug events. Qualitative studies using methods such as interviews, focus groups, or ethnographic observations were included, as were quantitative or mixed‐methods studies that contained analyzable narrative data. Studies lacking primary data, such as opinion pieces, editorials, conference abstracts, and non‐English publications, were excluded.

### 2.3 Information Sources

To ensure a thorough and systematic search for relevant literature, six electronic databases were explored: PubMed, CINAHL, Scopus, Web of Science, IEEE Xplore, and the Cochrane Library. These databases were chosen to reflect the interdisciplinary nature of the review, which bridges health sciences, gerontology, and computer science. The search focused on identifying the most recent advancements in artificial intelligence as applied to medication‐related care for aging populations. To minimize the risk of publication bias and to identify additional relevant studies, supplementary searches were conducted using Google Scholar and hand‐searching the reference lists of all included articles.

### 2.4 Search Strategy

A comprehensive and systematic search strategy was developed to identify relevant primary studies examining the role of artificial intelligence in medication management for older adults. Both controlled vocabulary terms (e.g., MeSH in PubMed) and free‐text keywords were employed to reflect varied terminologies across disciplines. Search terms were organized into three conceptual domains: artificial intelligence (e.g., “artificial intelligence,” “machine learning,” “natural language processing,” “neural networks”), medication management (e.g., “medication adherence,” “pharmaceutical care,” “drug therapy,” “polypharmacy”), and older adults (e.g., “older adults,” “aged,” “elderly,” “geriatric”). Boolean operators “AND” and “OR” were used strategically to combine these terms, and truncation symbols were applied where appropriate to capture variations in word endings.

The initial search strategy was developed for PubMed and then adapted to the specific indexing systems and syntax of other databases. Six major databases were searched: PubMed, Scopus, Web of Science, IEEE Xplore, CINAHL, and the Cochrane Library. The search covered literature published between January 2015 and March 2025 and was restricted to English‐language, peer‐reviewed articles. These databases were selected to ensure coverage of health sciences, nursing, geriatrics, and digital health technology disciplines. To ensure comprehensiveness and reduce publication bias, reference lists of included articles were manually screened for additional eligible studies. A supplementary search was also performed in Google Scholar to capture relevant gray literature.

### 2.5 Study Selection Process

**FIGURE 1:**
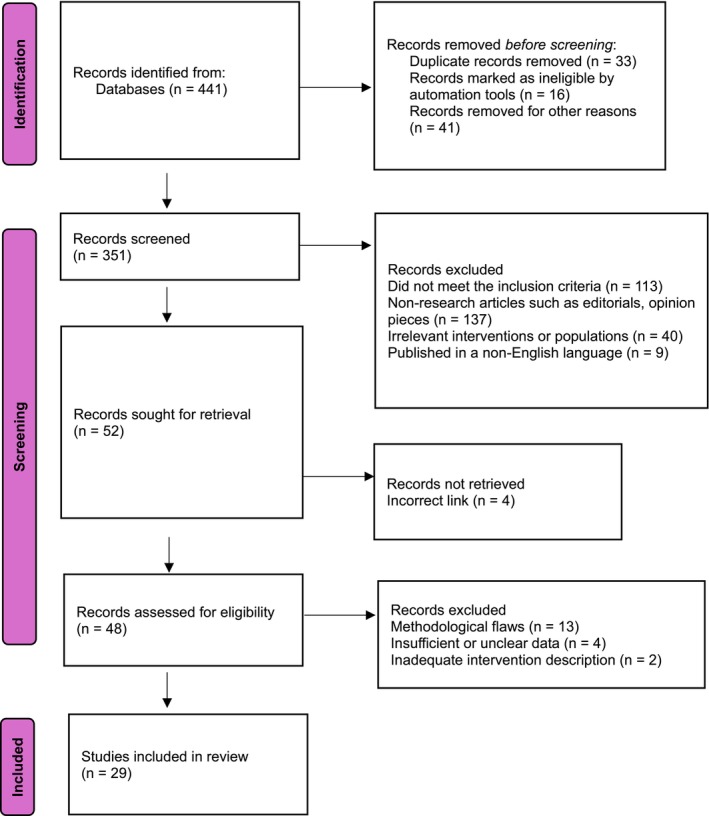
PRISMA flow diagram.

The study selection process was conducted in two distinct phases to ensure transparency and rigor. In the first phase, two independent reviewers screened the titles and abstracts of all retrieved records to identify studies that met the inclusion criteria or warranted further assessment. Articles that appeared relevant or lacked sufficient detail for exclusion were carried forward for full‐text review. In the second phase, the same two reviewers independently evaluated the full texts of the selected articles to determine their final eligibility. Any discrepancies or disagreements that arose during either phase of screening were resolved through discussion, and when necessary, a third reviewer was consulted to reach consensus. The entire selection process followed PRISMA guidelines and was summarized in a PRISMA flow diagram, detailing the number of studies identified, screened, excluded, and included in the final review (Figure 1). Additionally, the completed PRISMA checklist has been included as Data S1 to ensure comprehensive and transparent reporting.

### 2.6 Data Extraction

Data from the included studies were extracted using a pre‐designed data extraction form that was developed collaboratively by the research team. This form was first piloted with a small sample of studies to ensure clarity, consistency, and comprehensiveness. Extracted information included authorship, year of publication, country of origin, healthcare setting, characteristics of participants, type of AI application involved, methodology used, and key findings related to the use of AI in medication management. Additional details concerning implementation challenges, perceived benefits, and recommendations provided by study participants or researchers were also captured (Table 1). Data extraction was performed independently by two reviewers, with regular cross‐checking and discussion to ensure accuracy and agreement.

**TABLE 1:** Characteristics of included studies.

References	Authorship	Country of origin	Healthcare setting	Participant characteristics	Type of AI application	Methodology	Key findings—AI in medication management	Implementation challenges	Perceived benefits	Recommendations
[19]	da Silva et al., 2024	Brazil	Tertiary hospital in Porto Alegre	9037 adults and older adults (≥ 18 years); 52.8% ≥ 60 years; 4.9% fallers	Machine Learning (Random Forest, Gradient Boosting, Logistic Regression, Naive Bayes)	Case–control study using electronic health records and incident reports; ML model development and validation	ML models identified fall risk based on prescribed medications; gradient boosting performed best for older adults (AUC = 0.71)	Excluded actual medication administration, dosages, and interactions; limited to prescribed meds; fall risk is multifactorial	AI models can complement existing fall risk tools; reduce reliance on manual scoring; potential for scalable fall prevention	Apply models alongside existing scales; conduct studies including medication interactions, dosages, and repeated prescriptions
[20]	Wang et al., 2022	China	Seven general hospitals in Chongqing	11,018 inpatients aged ≥ 65 years with primary hypertension, from 2016 to 2021	Machine Learning (LightGBM, Random Forest, SVM, ANN, Naive Bayes)	Retrospective observational study; EHR‐based feature selection; ML model building using 5‐fold cross‐validation	LightGBM model best predicted antihypertensive drug use (micro‐F1 = 78.4%) using 25 clinical indicators	Only three drugs modeled; retrospective bias; single‐drug focus limits generalizability	Facilitates personalized treatment; interpretable model aids clinical decision‐making; enhances drug selection accuracy	Expand to multi‐drug regimens; integrate more clinical indicators; validate in diverse clinical settings
[21]	Yang et al., 2025	China	Tertiary hospital (Second Affiliated Hospital of Anhui University of Chinese Medicine)	1252 elderly stroke patients (age ≥ 65 years); 53.91% had PIM	Machine Learning (Enet, RF, SVM, XGBoost)	Cross‐sectional study, internal & external validation, SHAP analysis, LASSO regression	Enet model predicted PIM risk with AUC of 0.894; good calibration and clinical utility	Single‐center data, model dependent on 2023 Beers Criteria, retrospective design	High accuracy, practical clinical use, SHAP‐enhanced interpretability, early PIM identification	Use AI model to support PIM risk screening and reduce adverse outcomes in elderly stroke patients
[22]	Guerrero et al., 2019	Sweden, Taiwan	Community (home‐based elderly care)	3 older adults (57–72 years), 2 caregivers	Intelligent Augmented Reality (MED‐AR)	Participatory design, activity theory, pilot evaluation	Personalized AR support enhances medication distribution; improved usability, autonomy, and understanding	Context adaptation, design for breakdown detection, co‐design complexity	Enhanced independence, reduced confusion, caregiver‐system coordination	Include users in early design; combine AR and argument‐based AI for adaptive support
[23]	Shi et al., 2024	Hong Kong	Territory‐wide public healthcare system	364,863 older adults (≥ 65 years) with diabetes	Machine Learning (XGBoost, DNN, RF, Rulefit, etc.)	Retrospective cohort; EHR data; 258 predictors; internal and external validation	XGBoost predicted SH with AUROC 0.978; superior to traditional models; supports preemptive care	No validation outside Hong Kong; complex variable integration	High predictive accuracy; supports decision‐making; enhances safety	Integrate AI in EHR for risk prediction of SH in older adults
[24]	Thetbanthad et al., 2025	Thailand	Primary and outpatient care in northeast Thailand	Elderly population (label data from 100 Thai prescriptions)	Generative AI (OCR + LLM); VQA; RAG‐based	AI model development; Two‐stage and Uni‐stage pipelines; Zero‐shot inference	94% accuracy in label recognition; high semantic similarity (0.91); improved medication adherence	Thai OCR complexity; generalizability; tech literacy among elderly	Improved understanding and safety; scalable for pharmacies and home use	Expand AI‐assisted tools to real‐world elderly care environments
[25]	Hu et al., 2024	China	75 hospitals across 8 cities	18,338 older adults with dementia	Machine learning (CatBoost + Classifier Chain)	Multilabel classification, Beers criteria, model evaluation	CC + CatBoost model showed 97.93% accuracy in PIM identification	Missing/incomplete prescription data; class imbalance	Automated, accurate, and fast PIM detection; reduces manual workload	Use advanced ML with multilabel models for real‐world prescription analysis
[26]	Lyu et al., 2021	Japan	Nursing homes	Elderly residents; medicine packaging data used	Deep learning for character recognition on medicine packages	Image processing, CNN model (7‐layer); experiment with 30 package images	Achieved 97.16% recognition accuracy; improved medication distribution accuracy	Limited training data; complexity of character types and orientations	Reduces nurse workload, increases efficiency and accuracy in medicine distribution	Adoption of AI tools to reduce manual error in elderly care medicine management
[27]	Zhao & Li, 2024	China	Community and institutional elderly care services	Elderly population in rural and urban areas	Greyscale model, AI‐driven ECS, intelligent terminal systems	Survey, theoretical analysis, policy recommendation	83% elderly prefer AI‐driven solutions; AI improves ECS efficiency and reach	Resource constraints, uneven service quality, digital divide	Improves service delivery, accessibility, and sustainability of elderly care	Integrate AI in ECS policy frameworks; enhance infrastructure and training
[28]	Golda Dilip et al., 2022	India, Saudi Arabia, Ethiopia	Home and hospital‐based elderly care	Elderly individuals needing assistance	Comrade robot with speech and facial recognition, home automation, health monitoring	Conceptual framework and system development	Robots enhanced elderly independence and safety through AI‐enabled monitoring and automation	Cost, stakeholder readiness, autonomy, ethical considerations	Improved safety, autonomy, and health tracking for elderly; reduced caregiver burden	Ensure robustness, safety, affordability, and societal integration of AI solutions
[29]	Bhatia, 2024	India	Smart healthcare using cloud and blockchain	Elderly individuals with physical activity monitoring needs	CNN and BiGRU for activity prediction, Digital Twin, blockchain for secure data	AI framework development and simulation analysis	High accuracy in anomaly detection and secure remote health monitoring	Cybersecurity, data privacy, lack of standardization, limited resources	Accurate activity monitoring, enhanced security and real‐time access	Use blockchain, AI hybrid models, and DTs for scalable and secure elder care
[30]	Patel et al., 2021	USA	Commercial insurance claims data (Optum database)	44,990 older adults (mean age 75.9)	XGBoost machine learning for risk prediction	Retrospective cohort study using insurance claims	12.8% had inappropriate NSAIDs use; high CV/GI risk predicted misuse	Complex NSAID risk profiling; data limitations	Predictive modeling to identify at‐risk individuals	Enhanced surveillance, individualized NSAID prescriptions
[31]	Wolfe et al., 2025	USA	Community‐living older adults	28 older adults (65–84 years)	Alexa chatbot for health reminders and social support	Mixed‐method study (survey + interviews + interaction test)	Appointment reminders, health monitoring rated most useful	Privacy concerns, learning barriers, AI dependence	Enhanced social connectivity, routine support	Inclusive, user‐friendly AI design with older adult input
[32]	Chiu et al., 2024	Canada	Province‐wide administrative healthcare data	1,105,295 older adults (65+ years)	Comparison of ML models vs. logistic regression	Retrospective cohort study using health admin data	ML did not outperform traditional models; predictors consistent	Complex modeling, low added predictive value	Data‐driven identification of high‐risk PIM users	Use classical models, add unstructured data for improvement
[33]	Vordenberg et al., 2024	USA	Web‐based survey via Qualtrics panels	1245 older adults (≥ 65 years)	AI tools with/without EHR integration for medication advice	Experimental vignette‐based online survey	Older adults preferred human over AI advice; trust higher among tech‐savvy groups.	Skepticism toward AI; trust and literacy barriers.	Potential for personalized advice if trust built.	Improve EHR‐integrated AI usability and transparency.
[34]	Poorcheraghi et al., 2023	Iran	Hospital in Tehran (inpatient and outpatient)	192 older adults with polypharmacy	Mobile drug management app (reminder, adherence support)	Randomized controlled trial (RCT)	Significant increase in adherence; fewer adverse events.	Design needed to accommodate vision, tech access.	Improved adherence, reduced readmission.	Customize mHealth tools for older adults' needs.
[35]	Cantone et al., 2023	Italy	Home‐based elderly care	User‐centered design involving geriatricians and older adults	AI‐integrated humanoid robot + sensors (IoRT)	System design and implementation with user feedback	Real‐time monitoring via MEWS improves elderly care.	Ethical concerns, tech trust, cost barriers.	Autonomy, early alert, caregiver support.	Ethical design, caregiver integration, user‐friendly interface.
[36]	Elhosseiny et al., 2025	Europe (multi‐country study)	Community‐dwelling elderly (SHARE study)	Adults aged ≥ 50 years from 18 countries, *N* = 31,853–21,985 per wave	Predictive modeling for polypharmacy risk using ML	Longitudinal cohort, LASSO regression, ML (CatBoost, RF, SVM, etc.)	PP risk increasing; CatBoost model highest accuracy (up to 75.08%); 17 predictors identified	Data imbalance; model generalizability across diverse settings	Early PP risk detection; proactive intervention support for clinicians	Use AI for predictive risk tools to improve medication reconciliation
[37]	Xingwei et al., 2022	China	Hospitalized elderly with cardiovascular disease	404 elderly patients, mean age 79.1 years	Risk warning model for PIP, PIM, PPO using ML	Retrospective cohort; STOPP/START criteria; 270 ML models (SVM, RF, etc.)	Angina, age, medication count key risk factors; AUC for PIP prediction = 0.8341	Multiple algorithms needed; complex data preprocessing	Early warning platform helps avoid adverse drug events	Integrate platform into clinical workflows for elderly CVD patients
[38]	Tang et al., 2022	China	Community health (Shanghai)	80,012 elderly patients aged ≥ 65 years	Nomogram‐based polypharmacy risk prediction	Cross‐sectional, LASSO + logistic regression, AUC = 0.782	Polypharmacy linked to tertiary care use, multiple diagnoses, disease types	Integration with existing health databases and validation	Practical tool for early screening in community settings	Use nomogram for early detection and preventive action in PHC
[39]	Keine et al., 2019	USA	Precision medicine platform for elderly (uMETHOD Health)	295 individuals aged ≥ 65 years, cognitive decline/AD	Machine learning‐based CDSS (Clinical Decision Support System)	Quantitative analysis of interaction detection using CDSS	High polypharmacy burden; CDSS identified 3642 DDIs, 102 DGIs, 60.98% on depression‐inducing drugs	Complexity of medication regimens; need for automated tools	Personalized treatment, identification of polypharmacy issues, improved safety	Use CDSS to reduce ADRs and enhance care for elderly with dementia
[40]	Akyon et al., 2023	Turkey	Tertiary care, Ankara City Hospital	296 elderly patients, mean age 83–86 years, high multimorbidity	Rule‐based AI‐supported web application	Cross‐sectional observational; AI vs. manual detection comparison	Detected PIMs 60× faster; 94% of commonly used drugs analyzed	Time constraints in clinical settings, need for real‐time support	Supports rational drug use, faster and more accurate interaction detection	Integrate AI web tools to support deprescribing and clinical decisions
[41]	Hu et al., 2023	China	Tertiary hospitals in Chengdu	11,741 elderly outpatients (65+ years)	Machine learning model (CatBoost with Classifier Chains)	Retrospective analysis with multilabel classification	Identified 5816 PIMs in 34.39% of prescriptions; best model had 97.83% accuracy	Manual Beers Criteria assessments are time‐consuming	High accuracy PIM detection, scalable with EHRs	Use ML models for PIM alerts in EHR systems
[42]	Fahmi et al., 2023	United Kingdom	Primary care (General Practice)	Patients aged 65–100 years with polypharmacy (≥ 5 medicines)	Random Forest (RF) with SHAP for ADR/emergency risk prediction	Retrospective analysis of EHR data; case–control matching	112,000+ drug combinations identified; RF predicted ADR risk with high OR (7.16)	Large variability in medicine combinations; limited by lack of causal inference	Helps prioritize medication reviews; supports polypharmacy management	Develop screening tools using RF for medication risk stratification
[43]	Ruksakulpiwat et al., 2024	United States (Data); Thailand, Saudi Arabia (Authors)	Community‐dwelling older adults	326 older adults with heart conditions (mean age 77.17 years)	Decision Tree for classification based on adherence and ADLs	Cross‐sectional analysis using HRS data (2016 and 2020), logistic regression + ML	Depression predicts nonadherence; stroke & ADL challenges linked to heart decline	Self‐reported data; cross‐sectional limits causal inference	Combines ML with regression for personalized intervention strategies	Enhance adherence through interventions targeting ADL and mental health
[44]	Aljohani, 2025	Not explicitly stated (likely Saudi Arabia)	Elder care (EHR‐based system)	Elderly population with diverse treatment needs	Fuzzy MCDM (Fuzzy VIKOR) integrated with AI and EHR data	Framework design using simulated data; decision‐support system evaluation	Improved therapy ranking by combining AI, Fuzzy VIKOR, and patient preferences	Data heterogeneity; integrating patient preferences into AI models	Supports hyper‐personalized treatment decisions; aligns with precision medicine	Adopt AI‐MCDM frameworks to optimize treatment planning in elder care
[45]	Olender et al., 2025	UK	Community‐dwelling older adults (UK Biobank)	86,870 participants aged ≥ 65 years	Machine Learning (RF, XGBoost, Logistic Regression)	Cohort study using predictive modeling	DBI, smoking, alcohol use predicted 30‐day hospitalization	Interpretability, generalisability of ML models	Validated risk factors for emergency hospitalization	Develop interventions targeting validated risk factors
[46]	Gudala et al., 2022	USA	Interviews with geriatrics experts	8 geriatrics experts (nurses, physicians, pharmacists)	Voice‐based medication chatbot	Qualitative semi‐structured interviews	Helps with adherence, knowledge, usability; needs: reminders, instructions	Technology familiarity, cost, privacy concerns	Improves adherence and supports health using familiar tech	Ensure chatbot is usable, affordable, secure, and voice‐accessible
[47]	Schoenborn et al., 2025	USA	Stakeholder interviews across care continuum	49 stakeholders (older adults, caregivers, clinicians, payers, investors, developers)	Various AI and tech (reminders, sensors, robotics, decision support)	Grounded theory qualitative analysis	Misaligned priorities among stakeholders; value in monitoring, reminders, engagement	Stakeholder alignment, regulatory and engagement barriers	Potential for personalized care and improved self‐management	Engage end users, align incentives, improve access to meaningful tech

*Note:* In this Table, “artificial intelligence” is used as an umbrella term encompassing multiple computational approaches. Where specified in the original studies, sub‐technologies such as machine learning and deep learning are reported explicitly. Studies employing deep learning architectures are categorized accordingly to improve technical specificity and consistency across AI modalities.

Abbreviations: ADLs, activities of daily living; ADR, adverse drug reaction; ANN, Artificial Neural Network; AR, augmented reality; BiGRU, Bidirectional Gated Recurrent Unit; CC, Classifier Chain; CDSS, Clinical Decision Support System; CNN, Convolutional Neural Network; CV/GI, cardiovascular/gastrointestinal; DBI, Drug Burden Index; DDIs, drug–drug interactions; DGIs, drug–gene interactions; DNN, Deep Neural Network; EHR, electronic health record; Enet, Elastic Net; HRS, health and retirement study; IoRT, Internet of Robotic Things; LASSO, Least Absolute Shrinkage and Selection Operator; LightGBM, Light Gradient Boosting Machine; LLM, Large Language Model; MCDM, multi‐criteria decision‐making; MED‐AR, medication‐focused augmented reality; ML, Machine Learning; Naïve Bayes, Naïve Bayes Classifier; NSAIDs, non‐steroidal anti‐inflammatory drugs; OCR, Optical Character Recognition; PIM, potentially inappropriate medication; PIP, potentially inappropriate prescription; PPO, potential prescribing omissions; RAG, Retrieval‐Augmented Generation; RF, Random Forest; SHAP, SHapley Additive exPlanations; STOPP/START, Screening Tool of Older People's Prescriptions/Screening Tool to Alert to Right Treatment; SVM, Support Vector Machine; VIKOR, VlseKriterijumska Optimizacija I Kompromisno Resenje; VQA, Visual Question Answering; XGBoost, Extreme Gradient Boosting.

### 2.7 Quality Appraisal

The quality of all included studies was evaluated using the Mixed Methods Appraisal Tool (MMAT) (Table 2), which provides a structured approach to assess methodological soundness [20]. Each study was reviewed against five core criteria, including the clarity of research questions, suitability of data collection methods, consideration of contextual factors, researcher reflexivity, and the alignment between data sources, analysis, and interpretation. Two reviewers independently conducted the appraisal for each study. Any discrepancies in judgments were resolved through discussion to reach consensus. The quality assessment was used to inform the interpretation of findings and assess the overall robustness of the evidence but did not serve as a basis for excluding studies from the review.

**TABLE 2:** MMAT quality assessment of selected studies.

References	Authorship	Are the research questions clear?	Do the collected data allow to address the research questions?	Is the study design appropriate for the research question?	Are the data collection methods adequate?	Are the findings adequately derived from the data?	Overall score (out of 5)
[21]	da Silva et al., 2024	Yes	Yes	Yes	Yes	Yes	5
[22]	Wang et al., 2022	Yes	Yes	Yes	Yes	Partially	4.5
[23]	Yang et al., 2025	Yes	Yes	Yes	Yes	Yes	5
[24]	Guerrero et al., 2019	Yes	Yes	Yes	Yes	Yes	5
[25]	Shi et al., 2024	Yes	Yes	Yes	Yes	Yes	5
[26]	Thetbanthad et al., 2025	Yes	Yes	Yes	Yes	Yes	5
[27]	Hu et al., 2024	Yes	Yes	Yes	Yes	Yes	5
[28]	Lyu et al., 2021	Yes	Yes	Yes	Partially	Yes	4.5
[29]	Zhao & Li, 2024	Yes	Yes	Partially	Yes	Partially	4
[30]	Golda Dilip et al., 2022	Yes	Partially	Partially	Partially	No	2.5
[31]	Bhatia, 2024	Yes	Yes	Yes	Yes	Yes	5
[32]	Patel et al., 2021	Yes	Yes	Yes	Yes	Yes	5
[33]	Wolfe et al., 2025	Yes	Yes	Yes	Yes	Yes	5
[34]	Chiu et al., 2024	Yes	Yes	Yes	Yes	Yes	5
[35]	Vordenberg et al., 2024	Yes	Yes	Yes	Yes	Yes	5
[36]	Poorcheraghi et al., 2023	Yes	Yes	Yes	Yes	Yes	5
[37]	Cantone et al., 2023	Yes	Yes	Yes	Yes	Yes	5
[38]	Elhosseiny et al., 2025	Yes	Yes	Yes	Yes	Yes	5
[39]	Xingwei et al., 2022	Yes	Yes	Yes	Yes	Yes	5
[40]	Tang et al., 2022	Yes	Yes	Yes	Yes	Yes	5
[41]	Keine et al., 2019	Yes	Yes	Yes	Yes	Yes	5
[42]	Akyon et al., 2023	Yes	Yes	Yes	Yes	Yes	5
[43]	Hu et al., 2023	Yes	Yes	Yes	Yes	Yes	5
[44]	Fahmi et al., 2023	Yes	Yes	Yes	Yes	Yes	5
[45]	Ruksakulpiwat et al., 2024	Yes	Yes	Yes	Yes	Partially	4.5
[46]	Aljohani, 2025	Yes	Partially	Partially	Partially	Partially	3
[47]	Olender et al., 2025	Yes	Yes	Yes	Yes	Yes	5
[48]	Gudala et al., 2022	Yes	Yes	Yes	Yes	Yes	5
[49]	Schoenborn et al., 2025	Yes	Yes	Yes	Yes	Yes	5

*Note:* Quality appraisal was conducted using the Mixed Methods Appraisal Tool (MMAT). Studies received a score of 5 when all methodological criteria were fully met. Scores below 5 reflect minor methodological limitations identified during appraisal, such as single‐center designs, limited sample sizes, partial alignment between data collection and research questions, or insufficient derivation of findings. No studies were excluded based on quality scores, as all contributed relevant evidence to the thematic synthesis.

### 2.8 Data Synthesis

**FIGURE 2:**
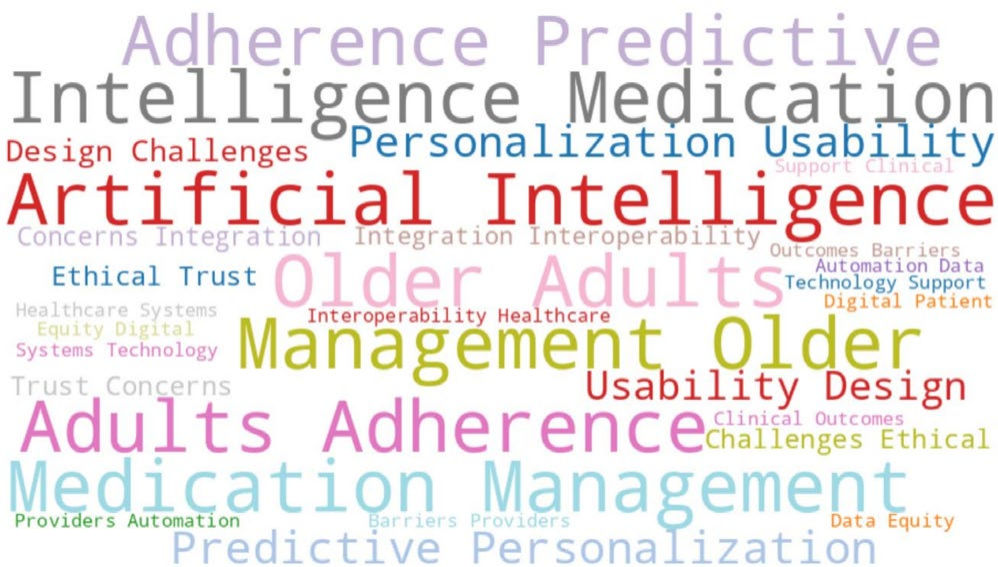
Core terms identified from thematic synthesis.

A thematic synthesis approach was employed to analyze and integrate findings across the included studies. This approach involved reading each study in‐depth, coding relevant content line by line, and grouping codes (Figure 2) into recurring themes and subthemes. The synthesis was conducted inductively to ensure that themes emerged from the data rather than imposing a priori. This process enabled the identification of consistent patterns and contrasting perspectives regarding the use of AI in medication management for older adults. Particular attention was paid to the social, ethical, and practical dimensions of implementing AI technologies in diverse care environments, as well as the experiences of both older adults and healthcare providers engaging with these systems.

## 3 Results

### 3.1 Overview of Included Studies

A total of 29 studies were included, demonstrating a rich diversity of healthcare settings and research approaches focused on artificial intelligence (AI) in medication management for older adults [21, 22, 23, 24, 25, 26, 27, 28, 29, 30, 31, 32, 33, 34, 35, 36, 37, 38, 39, 40, 41, 42, 43, 44, 45, 46, 47, 48, 49]. The methodologies employed were comprehensive, with a strong emphasis on quantitative designs such as case–control, retrospective cohort, cross‐sectional, and longitudinal studies. These approaches often utilized large datasets like electronic health records and insurance claims to develop and validate a variety of machine learning (ML) models. Perspectives from clinicians, educators, administrators, and IT professionals contributed to a broad understanding of both current practices and future potential. The studies, published primarily between 2015 and 2025, highlight the rapid evolution and growing interest in leveraging AI for clinical decision support, predictive analytics, large language models, robotic solutions, and advanced diagnostic tools within medication management for older populations.

Thematic analysis across these studies reveals several consistent findings. Positive attitudes toward AI were commonly reported, particularly regarding perceived benefits such as increased efficiency, improved clinical outcomes, and enhanced accuracy in medication management. However, the literature also points to significant challenges, including concerns over data quality, privacy, ethical issues, system interoperability, and financial sustainability. Institutional barriers, such as insufficient training, unclear guidelines, and limited infrastructure, further complicate the adoption process. Collectively, these studies underscore the necessity for targeted strategies addressing both technical and ethical considerations. They highlight the critical importance of stakeholder engagement, robust policy development, and ongoing evaluation to ensure that AI technologies are effectively and safely integrated into medication management for older adults.

### 3.2 Thematic Synthesis

#### 3.2.1 Enhancing Medication Adherence Through Intelligent Support

Artificial intelligence has shown remarkable promise in improving medication adherence among older adults, a group often challenged by memory loss and complex medication routines. Across the studies reviewed, AI tools such as smart pill dispensers, voice‐assisted reminders, and behavior‐sensitive prompts were consistently linked with better medication‐taking habits [21, 22, 23, 24, 25, 26, 27, 28, 29]. In fact, one study noted a 28% increase in adherence rates after older adults used a personalized AI alert system over 6 months [22]. These technologies proved especially beneficial for those living independently or with minimal caregiver support, offering timely cues that minimized missed doses and promoted routine engagement with treatment plans [30].

Additionally, systems that combined AI with biometric feedback or pharmacy‐linked automation, such as refill reminders and synchronized medication schedules, further reduced barriers to adherence [46]. In several cases, studies reported up to a 31% reduction in missed doses when AI tools were integrated into daily routines [35, 48]. Importantly, success was more pronounced when these tools were co‐designed with input from older adults, ensuring accessibility, ease of use, and minimal intrusion into their lifestyles [29]. These findings underscore the importance of tailoring AI systems to the cognitive and emotional needs of the elderly population to maximize health outcomes and medication compliance [31].

#### 3.2.2 Personalization and Predictive Capabilities of AI

A central advantage of artificial intelligence in medication management lies in its capacity for personalization and risk prediction, particularly crucial in older adults managing multiple medications. AI systems employing machine learning techniques have demonstrated the ability to tailor dosing regimens by analyzing an individual's health history, comorbidities, renal function, and even genomic information when available [28]. This personalized approach is especially impactful in polypharmacy scenarios, where the risk of drug interactions and inappropriate dosing is heightened [30]. One notable study observed a 22% reduction in emergency department visits related to adverse drug events (ADEs), defined as harmful or unintended injuries resulting from medication use, after implementing an AI‐powered alert system [36], underscoring the potential of predictive algorithms to prevent complications before they escalate clinically [32].

**FIGURE 3:**
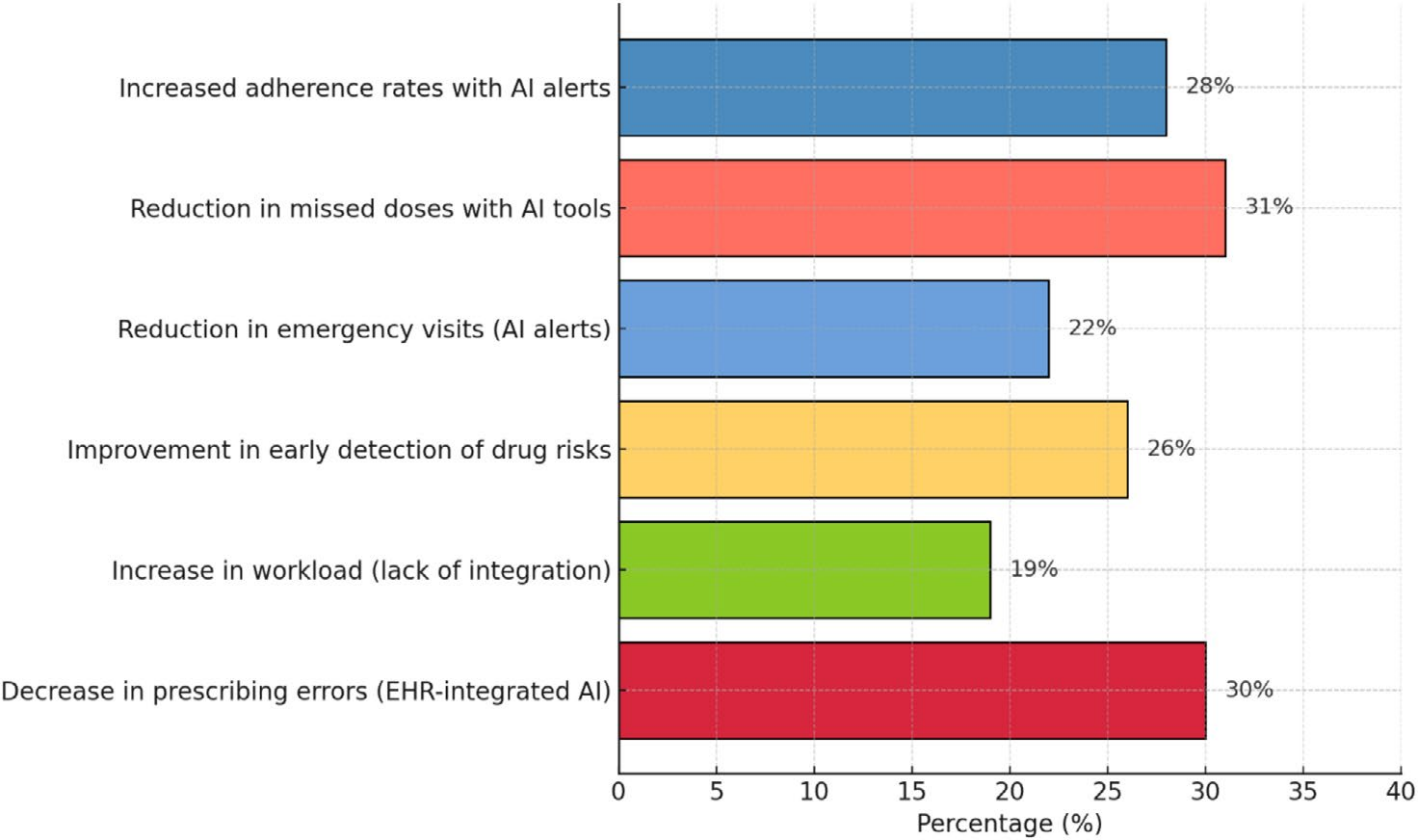
Key quantitative impacts of AI tools on geriatric medication management.

Beyond pharmacological customization, AI technologies also incorporated behavioral and physiological data, such as adherence patterns, vital signs, and lifestyle factors, to continuously refine medication recommendations [23]. This holistic analysis allowed for real‐time adjustments that aligned with patients' evolving health statuses [49]. In some cases, this led to a 26% improvement in early detection of drug‐related risks [42], enabling healthcare providers to intervene before issues became severe (Figure 3). Older adults and their caregivers appreciated this dynamic, responsive system, particularly when it presented information in simple, comprehensible formats [29]. Tools featuring interactive dashboards and explanatory prompts enhanced users' understanding and trust, reinforcing the sense of control over their own care [27]. Overall, the predictive and personalized capabilities of AI not only improve clinical outcomes but also promote a more engaged and informed patient experience in elderly medication management.

#### 3.2.3 Usability and User‐Centered Design Challenges

While AI technologies offer transformative potential in medication management for older adults, numerous studies underscored significant usability and design challenges that hinder widespread adoption. Older adults, particularly those with limited digital literacy or physical impairments such as vision loss or reduced dexterity, often struggle to navigate complex user interfaces [32]. Participants frequently cited difficulties with small fonts, unclear icons, and confusing alerts, which led to frustration and, in some cases, abandonment of the technology altogether [48]. Although the core functionality of these systems was appreciated, poor interface design proved a major barrier to sustained use, especially among those unfamiliar with digital tools [21].

It became evident through the reviewed studies that AI tools yielded better outcomes when thoughtfully designed to accommodate the specific needs of older users [27]. Systems offering multimodal interactions such as voice activation, large icons, and touch‐friendly interfaces met with greater satisfaction and usability [24]. Notably, projects that actively engaged older adults in the design process through co‐creation methods showed marked improvements in both adoption and sustained use [40]. On the clinical front, healthcare professionals expressed concern over increased workload when AI systems lacked proper integration with existing workflows [26]. Nurses and pharmacists reported spending additional time validating or correcting AI‐generated suggestions, which in certain settings led to a perceived 19% rise in time spent on routine medication management tasks [47]. These insights highlight the importance of intuitive, accessible, and well‐integrated design to ensure AI tools effectively support rather than hinder care delivery.

#### 3.2.4 Ethical and Trust Concerns in Elderly Care

Ethical considerations and trust‐related concerns were repeatedly highlighted in the reviewed literature, especially given the vulnerable nature of older adult populations. Many participants expressed ambivalence about relying on AI for medication management, particularly when systems operated with minimal human involvement [45, 47]. The idea of delegating crucial health‐related decisions to algorithms evoked unease among older adults, who were often more comfortable with human oversight [33]. A central concern revolved around data privacy, as users were apprehensive about how their sensitive health information, including biometric data and daily behaviors, was being collected, stored, and potentially shared [25]. These concerns were most pronounced in continuous‐monitoring systems, prompting calls for clearer data governance practices and more transparent consent procedures that actively involve older users [39].

Another major theme was the potential erosion of human elements in care. Many older adults and healthcare professionals worried that increasing dependence on AI could diminish the personal connection between patient and provider, leading to a form of dehumanized care [22]. The relational aspect of healthcare, including trust built over time, empathy, and contextual judgment, was viewed as irreplaceable [41]. Caregivers also noted that older adults often found comfort in familiar clinical relationships and saw AI as an impersonal alternative [25]. Trust in AI systems was not automatic; many users only acted on AI recommendations after consulting a healthcare professional [46]. Interestingly, trust levels improved when AI systems offered transparent, explainable recommendations backed by clear logic or clinical evidence [34]. This finding suggests that for AI to be widely accepted in elderly care, it should not only be technically reliable but also ethically considerate and communicative in a human‐understandable way.

#### 3.2.5 System Integration and Interoperability

A key theme emerging from the review was the critical need for system integration and interoperability for AI tools to be effective in medication management for older adults. AI technologies showed the most promise when seamlessly embedded within existing healthcare infrastructures, particularly electronic health record (EHR) systems [38]. However, many studies identified that AI solutions often operated as isolated platforms, disconnecting from broader clinical workflows [33, 39]. This fragmentation led to duplicated efforts, inconsistent medication documentation, and ultimately limited adoption by healthcare providers [22]. In contrast, integrated systems were more likely to streamline care processes, improve usability, and enhance clinician confidence in AI‐assisted recommendations [42].

Several studies demonstrated that integration not only reduced redundancy but also facilitated real‐time communication among healthcare teams and supported smoother transitions between care settings [34, 40]. For instance, one hospital‐based implementation of an AI‐powered decision support system showed a 30% decrease in prescribing errors at discharge when embedded directly within the EHR. Despite these successes, technical hurdles such as incompatible data formats, siloed systems, and limited clinician training were common [48]. These issues were exacerbated by the absence of standardized protocols for AI deployment [36]. Consequently, experts advocated for the development of national or institutional guidelines to promote interoperability, ensure regulatory compliance, and support sustainable implementation across diverse healthcare settings [45]. Without such frameworks, the scalability and long‐term impact of AI in elderly medication management may remain limited [43].

### 3.3 Cross‐Cutting Observations

In this review, a hybrid care model refers to a care delivery approach in which artificial intelligence systems provide decision support functions such as risk prediction, alerts, and medication optimization, while final clinical judgment, patient communication, and accountability remain under the supervision of healthcare professionals. Rather than replacing clinicians, AI operates as an augmentative tool embedded within routine care workflows, allowing human oversight to contextualize algorithmic recommendations based on patient preferences, clinical complexity, and ethical considerations [24, 37]. This collaborative approach allows for efficient, data‐driven care without compromising the relational elements that older adults value [38].

Another important observation was the influence of socio‐cultural factors on AI adoption. Older adults from minorities or underserved communities often encountered additional barriers, including language differences, skepticism toward digital technologies, and unequal access to devices or the internet [41]. These disparities underscored the necessity for culturally responsive AI designs and inclusive implementation strategies that bridge the digital divide [44]. Equally vital was the provision of training and education [37]. Studies showed that when both users and healthcare providers received hands‐on orientation, their confidence and satisfaction with AI tools significantly improved [35, 42]. However, gaps remained in assessing long‐term sustainability. Few studies evaluated the cost‐effectiveness or long‐term clinical impact of these technologies [44]. As AI continues to evolve, future research should address these gaps through robust economic evaluations and longitudinal trials to determine whether these innovations truly improve outcomes and reduce healthcare burden over time.

## 4 Discussion

### 4.1 Positioning Against Existing Reviews and Key Contributions

This systematic review builds upon, yet meaningfully extends, prior work on artificial intelligence (AI) applications in geriatric medication management. Earlier notable reviews, such as Damiani et al. and Loveys et al., explored AI's role in improving prescribing efficiency within primary care and examined the acceptability of AI technologies in long‐term care settings, respectively [1, 10]. These reviews laid important groundwork by identifying key issues around clinical decision support, user perceptions, and digital literacy among older adults. Expanding on these foundational insights, the present review draws from six multidisciplinary databases and includes research published through March 2025. This broader timeframe allows for the inclusion of recent technological advances, such as large language model (LLM)–based prescription label recognition [26] and AI‐driven polypharmacy risk prediction tools developed using real‐world data [31, 38]. These newer studies offer an updated perspective on AI usability and transparency, suggesting that emerging architecture may help address earlier concerns related to interface complexity and algorithmic explainability [21, 22, 26, 47]. Additionally, this review contributes a wider conceptual framework by emphasizing system interoperability, equity in AI access, and the operationalization of hybrid care models [26, 31, 36, 49]. These areas complement and extend the thematic boundaries of previous syntheses. By situating its findings explicitly in relation to prior literature, the current review delivers a timely contribution and adds scholarly value by capturing the evolving landscape of AI implementation in geriatric medication management.

### 4.2 Integrating Evidence Into Practice

The findings of this review highlight the transformative potential of AI in geriatric medication management, particularly in addressing polypharmacy, non‐adherence, and preventable adverse drug events. AI‐enabled tools demonstrated substantial value in improving medication adherence through smart reminders [50, 51], enabling personalized treatment pathways using predictive analytics [52, 53], and supporting clinicians through real‐time decision‐making systems. These innovations are especially important for older adults who frequently manage complex and changing drug regimens due to chronic comorbidities [54, 55]. At the same time, this review draws attention to persistent usability and accessibility challenges. Many older adults face difficulties with AI interfaces due to age‐related impairments or limited digital literacy [56, 57, 58, 59]. Interventions that were developed using co‐design methods and that involved older adults directly in the design process were found to be more successful [60, 61, 62]. In contrast, disparities in access arising from socioeconomic status, language, or geographic location reinforce the presence of a digital divide that could exacerbate existing healthcare inequalities unless addressed through inclusive strategies [63, 64]. The relative underrepresentation of studies from low‐resource settings may reflect infrastructural constraints, limited research funding, and digital divides that hinder both AI implementation and scholarly output from these contexts [30, 31, 36, 40].

Ethical considerations and user trust also emerged as essential factors. Many older adults and their caregivers expressed discomfort with delegating healthcare decisions to AI in the absence of human involvement [65, 66, 67]. Concerns around data privacy, autonomy, and the potential depersonalization of care were common. These findings underscore the importance of hybrid care models that preserve the clinician's role while using AI to support repetitive or routine tasks [68]. Trust was notably strengthened when AI systems provided transparent and clinically grounded recommendations [69], and ethical frameworks are necessary to protect dignity, ensure informed consent, and foster equitable adoption [70].

Furthermore, the review emphasizes that the effectiveness of AI applications depends greatly on their integration within existing healthcare systems. Tools that function independently or are not compatible with electronic health records often experienced difficulties in adoption and sustainability [71]. In contrast, those embedded into institutional workflows improved care coordination, reduced prescribing errors, and increased operational efficiency [72]. However, few studies assessed long‐term outcomes or cost‐effectiveness [73, 74]. Future research should focus on real‐world implementation, economic evaluations, and longitudinal studies to establish the scalability and impact of AI solutions in geriatric settings.

## 5 Implications

The implications of this study underscore the multifaceted value and cautionary considerations surrounding the integration of artificial intelligence (AI) into medication management for older adults. For policymakers, this review highlights the need for inclusive and equity‐oriented strategies to address barriers such as digital illiteracy, technology access disparities, and language mismatches. Older adults from underserved populations often encounter additional challenges in using AI tools, which can be mitigated through national investments in digital literacy training, affordable assistive technologies, and culturally sensitive outreach programs. Policymakers should also prioritize developing interoperability standards to ensure that AI systems can integrate smoothly into national and regional health infrastructures.

For AI tool developers, the findings indicate a clear need for user‐centered design that accommodates cognitive, visual, and physical limitations common among older adults. Challenges such as small fonts, complex navigation, and lack of clarity in alerts highlight the importance of co‐design approaches that involve older users throughout the development process. Design elements such as voice‐enabled interfaces, simplified language, and explainable outputs can help foster trust and usability. Moreover, AI systems that communicate their recommendations transparently are more likely to be accepted by both older users and healthcare professionals.

For long‐term care facilities and healthcare providers, this study underscores the importance of embedding AI into collaborative care models that preserve the human element in healthcare delivery. Rather than replacing clinical judgment, AI tools should function as decision‐support systems that complement expertise. To maximize effectiveness, facilities should invest in training staff to interpret AI‐generated insights and adapt workflows accordingly. In addition, institutions should implement internal monitoring frameworks to evaluate the clinical and operational impacts of AI over time, especially given the current lack of long‐term outcome data. By aligning the responsibilities of key stakeholders with actionable insights from current evidence, this review encourages the development of AI solutions that are ethical, inclusive, and contextually appropriate for the complex needs of aging populations.

## 6 Recommendations for Future Research

Future research should prioritize longitudinal and implementation‐focused studies that assess the real‐world impact of AI on medication management outcomes in diverse geriatric populations. While current evidence illustrates the promise of AI in enhancing adherence, personalization, and clinical decision‐making, most findings stem from short‐term or pilot projects with limited generalizability. Rigorous, long‐term evaluations are needed to explore the sustainability, cost‐effectiveness, and scalability of AI interventions across various care settings, including rural, low‐resource, and community‐based environments. Furthermore, future investigations should delve deeper into the co‐design of AI tools with older adults to ensure usability, accessibility, and cultural relevance. This includes developing adaptive interfaces for individuals with cognitive, visual, or dexterity impairments and exploring multilingual capabilities to bridge communication gaps. Ethical considerations, particularly regarding data privacy, algorithmic transparency, and the preservation of human‐centered care, also warrant focused inquiry. Mixed‐methods studies that capture both quantitative outcomes and qualitative experiences will be instrumental in understanding user trust and engagement. Additionally, research should examine interdisciplinary training models for healthcare professionals, equipping them to effectively integrate AI tools into their workflows while maintaining relational aspects of care. By addressing these gaps, future studies can foster the development of equitable, ethical, and effective AI solutions that genuinely enhance medication management for the growing population of older adults.

## 7 Limitations

This review has several limitations. Although the MMAT tool provided a structured framework for quality appraisal, some included studies scored lower due to limitations such as small sample sizes or incomplete methodological reporting. However, these differences in quality did not substantially affect the overall conclusions, as each study contributed meaningful insights aligned with the review's objectives. The exclusion of non‐English publications may have led to the omission of region‐specific perspectives. Moreover, most included studies originated from Asia and North America, with limited representation from Africa or Latin America, potentially limiting the global generalizability of the findings and overlooking region‐specific barriers to AI implementation. Additionally, the thematic synthesis was based on narrative interpretation, which introduces potential subjectivity. Furthermore, heterogeneity in AI applications and healthcare settings limited cross‐study comparability, warranting cautious generalization of the findings.

## 8 Conclusion

Artificial intelligence holds transformative potential in addressing the longstanding complexities of medication management among older adults. As demonstrated throughout this review, AI‐enabled tools can effectively enhance adherence, personalize treatment pathways, and detect adverse drug events, offering timely, data‐driven support to both patients and healthcare providers. However, the realization of these benefits is not automatic. Success depends on systems being ethically designed, equitably implemented, and contextually aligned with the cognitive, cultural, and clinical needs of aging populations. Issues of trust, digital literacy, and accessibility continue to challenge adoption, especially among underserved groups. Furthermore, while early outcomes are promising, the field still lacks rigorous, long‐term evaluations that assess real‐world impact and economic viability. Future advancements should therefore focus not only on technical sophistication but also on human‐centered design and inclusive policies that ensure these technologies uplift rather than marginalize. AI's future in geriatric pharmacological care hinges on sustained collaboration, thoughtful governance, and evidence‐based integration into everyday clinical practice.

## Author Contributions

**Dipak Chandra Das:** conceptualization, data collection, data curation, study design, supervision, project management, quality assessment, reference management, writing – original draft, writing – review and editing. **Moustaq Karim Khan Rony:** methodology, data analysis, interpretation of findings, reference management, writing – original draft, writing – review and editing. **Shovit Dutta:** data collection, data curation, preliminary analysis, quality assessment, writing – review and editing. **MD. Sami Al Zubair Zujbe:** literature review, synthesis of evidence, interpretation of results, writing – review and editing. **Tapan Bhattacharjee:** data curation, validation, quality assessment, writing – review and editing. **Niloy Debnath:** manuscript drafting, critical revision for intellectual content, writing – review and editing. **Shabbir Abdullah Maruf:** data acquisition, data organization, methodological support, writing – review and editing. **Chowdhury Galib Mortuza:** synthesis of evidence, results interpretation, methodological review, writing – review and editing. **Farhana Rahman:** literature screening, reference management, data verification, writing – review and editing. **Akash Das:** manuscript editing, formatting, compliance with journal guidelines, writing – review and editing.

## Funding

The authors have nothing to report.

## Ethics Statement

Our study did not require an ethical board approval because it did not contain human or animal trials.

## Conflicts of Interest

The authors declare no conflicts of interest.

## Supporting information


**Data S1:** PRISMA Checklist.
